# Facile synthesis of nanosized Mn_3_O_4_ powder anodes for high capacity Lithium-Ion battery *via* flame spray pyrolysis

**DOI:** 10.3389/fchem.2022.990548

**Published:** 2022-08-11

**Authors:** Hao Wang, Jiachang Zhao, Dongmei Xie, Haiji Huang, Pinhua Rao, Jianfeng Mao

**Affiliations:** ^1^ School of Chemistry and Chemical Engineering, Shanghai University of Engineering Science, Shanghai, China; ^2^ School of Chemical Engineering and Advanced Materials, The University of Adelaide, Adelaide, SA, Australia; ^3^ Institute for Superconducting & Electronic Materials, University of Wollongong, Wollongong, AU-NSW, Australia

**Keywords:** spray pyrolysis, Mn3O4, nanopowder, lithium-ion battery, anode

## Abstract

Mn_3_O_4_ powders with nanometer size are successfully synthesized by a simple one-step method via flame spray pyrolysis. The precursor droplet is generated by heating under high temperature flame with fixed flow rate, and the exothermic reaction is induced to form nanosized Mn_3_O_4_ powders. When used as anode material for lithium-ion battery, the Mn_3_O_4_ exhibits good cycling capacity and rate performance. It delivers a specific capacity of 1,182 mA h g^−1^ over 110 cycles at a current density of 200 mA g^−1^, and has a high capacity of 140 mA h g^−1^ at 5,000 mA g^−1^.

## Introduction

Lithium-ion battery (LIB) is widely used in people’s daily life in portable electrical appliances such as mobile phones, cameras, and notebook computers, and is also rapidly expanding to the field of electric vehicles. This is due to the excellent performance of lithium-ion batteries compared with other batteries, such as high operating voltage (3.2–3.7 V), high specific energy (140 Wh/Kg), no memory effect, small self-discharge, and long cycle life, which makes it the most ideal power source in the 21st century. To satisfy the demands of electric vehicles and advanced electronics, the performance of LIB needs to be improved continuously. This leads to the significant demand for developing high performance electrode materials (Li et al., 2018; Mao et al., 2022; Wu et al., 2019). Transition metal oxides (TMOs) such as Co_3_O_4_, (Reddy et al., 2014; Wen et al., 2018) ZnO, Wang et al. (2018) CuO, (Huang et al., 2013; Reddy et al., 2013; Zhang et al., 2014; Zhang et al., 2014; Xu et al., 2016; Zhang et al., 2016) Fe_3_O_4_, (Ma et al., 2015; Petnikota et al., 2015; Li et al., 2019; Wang et al., 2019) Fe_2_O_3_ (Wang et al., 2015; Teng et al., 2018) and MnO_x_ have attracted widespread attention as promising anode materials for LIB due to their low cost and high theoretical capability. However, the electrode volume changes greatly during the lithiation/delithiation processes, which leads to the continuous and accumulated pulverization, resulting in rapid capacity decay.

In order to overcome the issues of transition metal oxides, many methods have been adopted to prepare transition metal oxides by modifying or controlling the morphology (Qiu et al., 2019; Liu et al., 2022; Zhao et al., 2022).

Among these methods, the development of nanostructured metal oxide materials has attracted extensive attention for improving the cycling and rate performance. The preparation of nanosized metal oxides has proven to be an effective strategy because the nanomaterials can better accommodate the strain of lithium ion insertion/extraction and provide a shorter path for lithium ion and electron transfer.

In recent years, manganese oxides have attracted much attention due to their high theoretical capacity, non-toxic, rich content and low cost. The preparation of manganese oxides with different stoichiometric components, such as manganese oxide: MnO_2_, Mn_2_O_3_, and Mn_3_O_4_, still has certain limitations in the rate performance and cycle stability, so it is still a challenge for researchers to develop new MnO_x_. (Deng et al., 2012; Wang et al., 2013; Wei et al., 2020) The method to synthesis special structure manganese oxides with high electrochemical performance is considered as a major research focus for lithium ion battery anode materials. At present, there are many methods to prepare various nanostructured materials, but wet phase-based methods require significant solvent costs, generate large amounts of waste water and require calcination steps after synthesis, adding to their costs.

Here, nanosized Mn_3_O_4_ powders were synthesized by flame spray pyrolysis as anode materials for LIB. The solution is a mixture of Mn(NO_3_)_2_ and sucrose. The droplets formed in the high temperature of the flame are dried and dehydrated to form Mn_3_O_4_ powder with nanometer size. These Mn_3_O_4_ nanoparticles demonstrated high reversible capacity of 1,182 mA h g^−1^ at 200 mA g^−1^ over 110 cycles and retained a capacity of 140 mA h g^−1^ at a high current density of 5,000 mA g^−1^.

## Experimental section

### Materials

Mn(NO_3_)_2_·4H_2_O, sucrose (C_12_H_22_O_11_), and hydrogen peroxide (MW:30%wt) were purchased from Sigma-Aldrich.

### Synthesis of nanosized Mn_3_O_4_ powders via flame spray pyrolysis method

**FIGURE 1:**
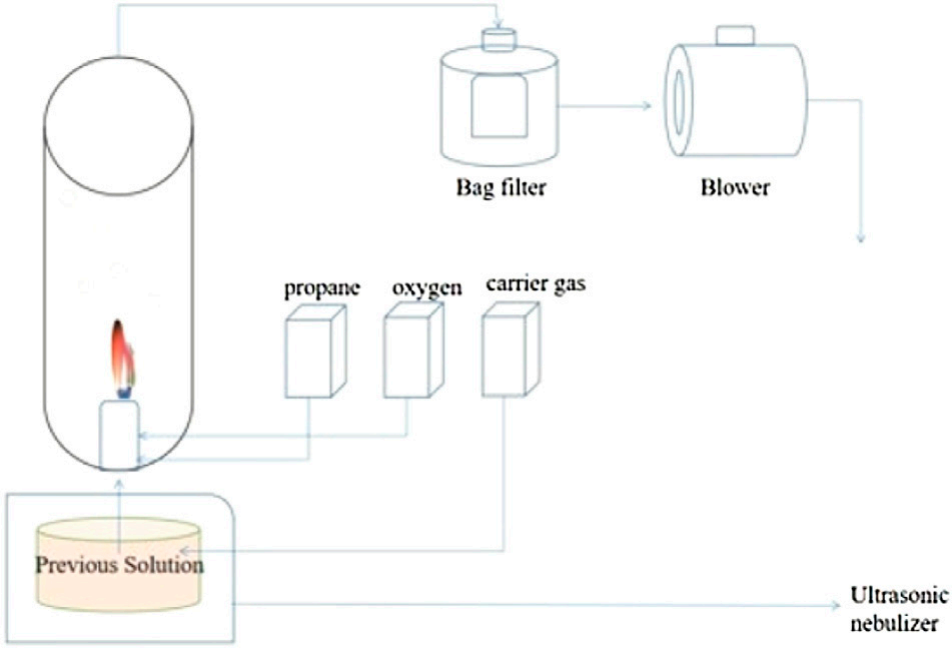
Schematic of the flame spray pyrolysis process.

To fabricate the nanosized Mn_3_O_4_, a flame spray pyrolysis method was employed as illustrated in Figure 1. 0.4 M Manganese nitrate with 0.5 M sucrose and 5 ml hydrogen peroxide solution were mixed in 15 ml distilled water as the precursor, among which sucrose and hydrogen peroxide were the blowing agents. The flow rates of the fuel, oxidizer, and carrier gas of the flame spray pyrolysis system were fixed at 5, 40, and 10 L min^−1^, respectively.

### Assembly of the batteries

To prepare the electrode, the nanosized Mn_3_O_4_ powder materials, carbon black and sodium carboxymethyl cellulose (CMC) binder were mixed together at a weight ratio of 7.5:1.5:1.5 in water to form a slurry and was pasted onto a piece of Cu foil using a doctor blade. The electrode was vacuum-dried at 100°C for 6 h, rolled under pressure, and then punched to match the desired size of a CR2032 coin cell. The active material loading was 0.8 (±0.2) mg cm^−1^. Coin cells were assembled, with lithium foil as counter electrode, 1 M LiPF_6_ in a mixture of ethylene carbonate/diethyl carbonate (EC/DEC, volume 1/1) as electrolyte, and Selgard ®2,400 (Selgard, LLC, United States) as the separator.

## Characterization

### X-Ray diffraction

X-ray diffraction (XRD) data were collected on a Rigaku Smartlab SE using Cu Kα radiation.

### Scanning electron microscope

The morphology of nanosized powders was imaged on a FEI Scios 2 HiVac scanning electron microscope (SEM). The accelerated voltage was 15 kV. And the electrode material after 110 cycles of charge/discharge at a density of 200 mA g^−1^ was investigated.

### Transmission electron microscope

The ultrastructure of the nanosized powder was imaged on a JEOL JEM-2100F transmission electron microscope (TEM).

### Electrochemical performance

Cyclic voltammetry (CV) was performed using a double potentiostat (CH, CHI760E) between 0 and 3.0 V with a potential sweep rate of 0.1 mV s^−1^. A Neware battery tester (CT-4008, China) was used to evaluate the electrochemical performance. Rate capability was examined at various current densities from 200 to 10,000 mA g^−1^.

## Results and disscussion

### Synthesis of the nanosized Mn_3_O_4_ materials: Reaction mechanism of flame spray pyrolysis and formation mechanism of nanoparticles

After the precursor solution is atomized, the droplet will undergo the following process: the solvent evaporates, the droplet diameter becomes smaller, the solute concentration on the droplet surface increases continuously and reaches the critical supersaturated concentration at a certain point, and the nucleation process will occur in the droplet. The result of nucleation is that the concentration anywhere in the droplet is less than the equilibrium concentration of the solute. After nucleation, the solvent in the droplet continues to evaporate, the droplet continues to decrease, and the mass percentage of solute in the droplet continues to increase. At the same time, the crystal nucleus also diffuses to the center of the droplet. With the further diffusion of crystal nuclei and the further reduction of droplet diameter, crystal nuclei with a certain size on the surface of the droplet contact with each other and solidified until they completely cover the droplet surface, then the droplet shell is formed. Thereafter, the droplet diameter does not change. After the shell is formed, the solvent in the droplet continues to evaporate, and the solute exceeding its equilibrium concentration precipitates on the crystal nucleus surface outside the droplet shell (excluding the shell), which promotes the growth of this part of crystal nucleus. If there is a crystal nucleus in the droplet center when the shell is formed, the generated particles are solid particles. If there is no crystal nucleus in the center of the droplet, the resulting particles are hollow particles. The particle shell is formed by the contact and solidification of crystal nuclei.

This process is realized by the flame spray pyrolysis of the precursor solution from “droplets to particles” in the high temperature process of flame heating, that is, the precursor solution which contains manganese metal salts, sucrose and H_2_O_2_, is atomized and thermally decomposed to form a hollow particle under the condition of low supersaturation under the condition of evaporation, chemical reaction and sintering. As is shown in Figure 1, the key to the formation of manganese oxide structure with small nanoparticles as components is to introduce sucrose and hydrogen peroxide (H_2_O_2_) into the initial solution of manganese nitrate as an *in situ* blowing agent.

### Morphology and phase analysis of nanosized Mn_3_O_4_ powder

**FIGURE 2:**
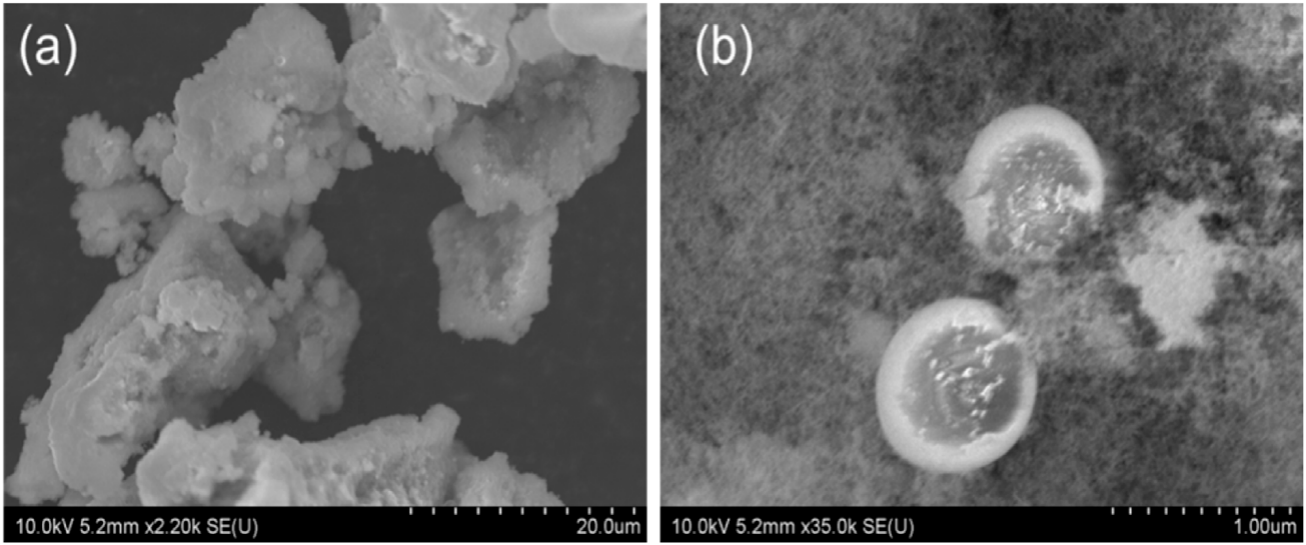
SEM images of the nanosized Mn_3_O_4_ powders: **(A)** low magnification,**(B)** higher manification.

**FIGURE 3:**
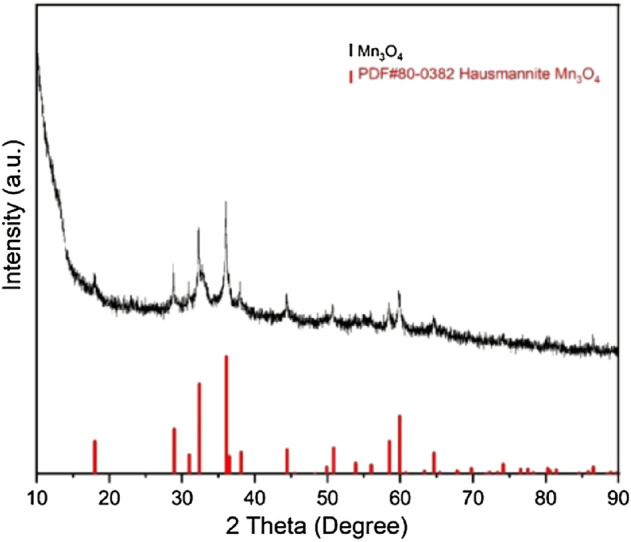
XRD pattern of nanosized Mn_3_O_4_ powders.

In order to have a deeper understanding of the morphology and substance equality of the synthesized material, we characterized the product by using SEM, XRD and TEM. From the Figure 2, we can see the SEM image of the morphologies of the nanosized Mn_3_O_4_ powders synthesized by flame spray pyrolysis method. At a low magnification, irregular powders can be seen in Figure 2A; At a higher magnification, the clear nanostructure was illustrated in Figure 2B. Some nano-sized spheres were also observed. Besides, Figure 3 is the X-ray diffraction pattern of the Mn_3_O_4_ material. All of the diffraction peaks can be indexed to the hausmannite (Mn_3_O_4_) tetragonal crystal structure (JCPDS file No. 80–0382) with lattice constants a = 5.762, b = 5.762, c = 9.470, which means good crystallinity and high purity of synthesized material.

**FIGURE 4:**
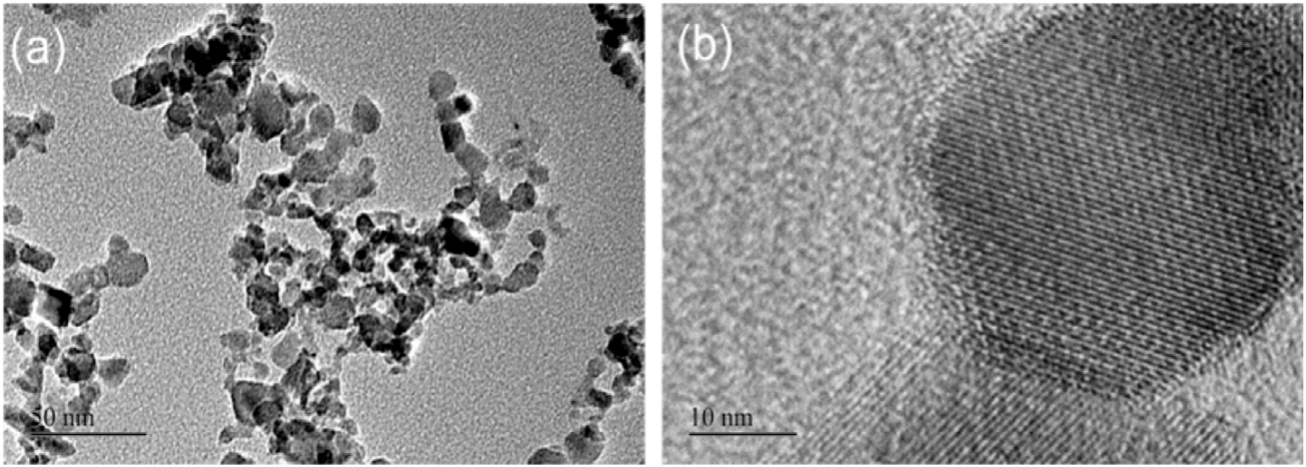
TEM images. **(A)** TEM image, **(B)** High-revolution TEM image.

In order to have a more detailed structure of the nanoscale powder. TEM test was performed on it and the image was illustrated in Figures 4A,B, the Figure 4A is the low resolution, which exhibited the various morphologies and nonuniform distribution of the Mn_3_O_4_ crystal structures. And the high resolution TEM image as shown in Figure 4B revealed the crystalline structure of the Mn_3_O_4_ nanosized powders, with ultra-thin walls. The sizes of Mn_3_O_4_ powders measured from TEM images vary from 10 to 30 nm.

### Electrochemical performance

**FIGURE 5:**
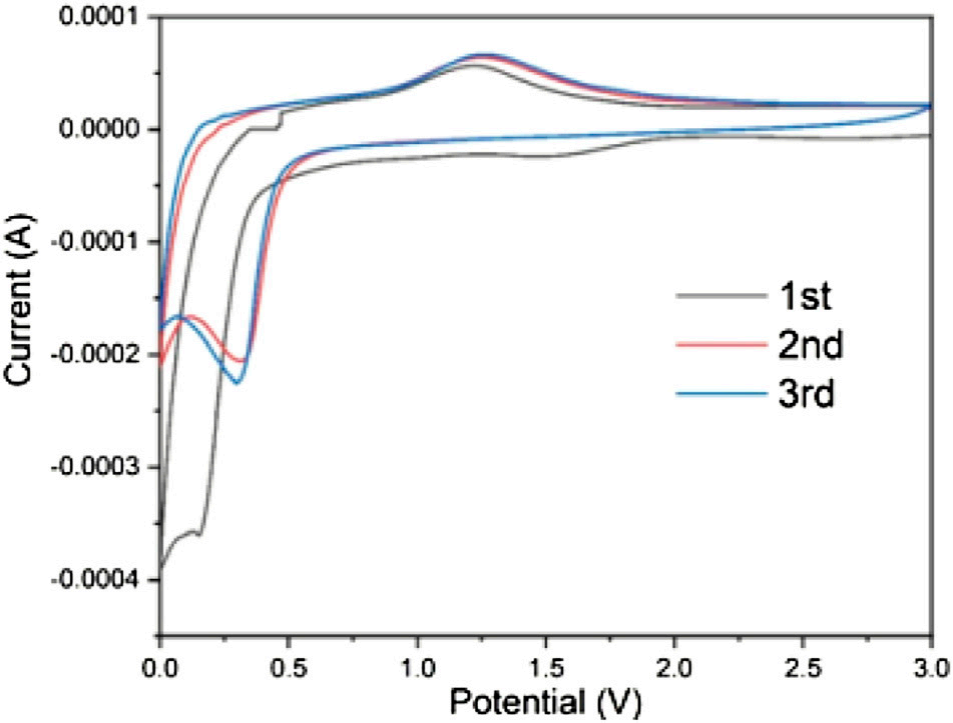
Cyclic voltammograms of the initial three cycles of Mn_3_O_4_. (scan rate 0.1 mV s^−1^

After the preliminary exploration of the physical properties of the material, the electrochemical properties of the material was studied. Figure 5 showed the CV curves in the initial 3 cycles of the nanosized Mn_3_O_4_ electrode materials between 0.0 and 3.0 V at a scan rate of 0.1 mV s^−1^. There was a wide reduction peak around 1.5 V in the first scan but disappeared on the second cycle. This is because of the decomposition of electrolyte and formation of solid-state interface films and the reduction of Mn^3+^ to Mn^2+^. In the first cycle, an obvious reduction reaction occurred near 0.15 V, and it shifted to 0.3 V in the following two cycles, corresponding to the reduction reaction to Mn^0^ and the formation of Li_2_O. The different reduction peak is a common feature of the manganese oxide electrode materials resulting from the structure changes of the lithium insertion process during the first cycle. During the anodic oxidation process, the oxidation of Mn^0^ to Mn^2+^ occurred at about 1.3 V, while a wide peak occurred after 2 V is allocated to Mn^2+^ for further oxidation to Mn^3+^. The results showed that the composite materials have good reversibility and stability.

**FIGURE 6:**
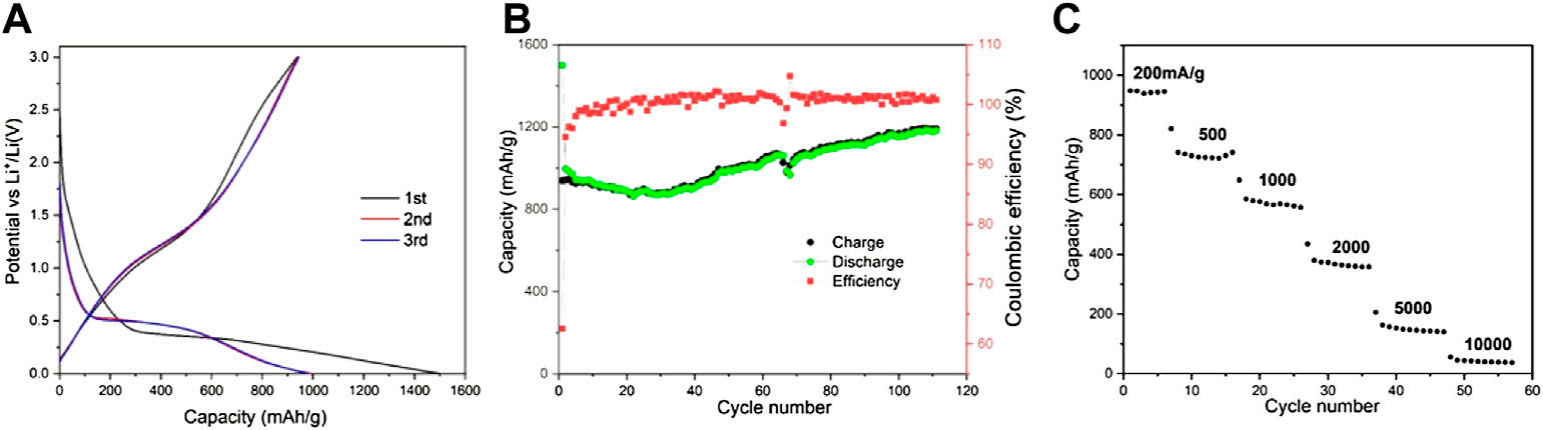
Electrochemical performance of nanosized Mn_3_,O_4_ as electrode materials: **(A)** Charge/discharge profiles for the initial three cycles at a current density of 200 mA g I, **(B)** Cycling performance at a current density of 200 mA g^−1^. **(C)** Rate performance at various current densities.

Figure 6A showed the charge and discharge curves of the nanosized Mn_3_O_4_ powders in the first three cycles. In the discharge curve of the first circle, the voltage curve above 0.4 V showed that it was caused by the formation of SEI film and the initial reduction of Mn_3_O_4_. It can be attributed to the irreversible electrochemical decomposition of the liquid electrolyte. The main lithiation reaction of the Mn_3_O_4_ spheres leads to the formation of voltage plateau at 0.4 V. However, from the second cycle, the platform shifted upward to a higher voltage (over 0.5 V), this is related to the structure changes as analyzed in the CV diagram above. The structural changed in the first cycle reduce the subsequent lithium resistance. In the charging process of the first cycle, there were two slope platforms in 1.0–1.5 V and 2.5–3.0 V, which can form Mn^2+^ and Mn^3+^ for the oxidation of Mn^0^. Gao et al. (2011)And in the first cycle, charge and discharge capacities came to 1,500 and 944 mA h g^−1^, respectively. The Coulombic efficiency for the second cycle is higher than 95%. The conversion reaction: Mn_3_O_4_ + 8Li^+^ + 8e^−^→3Mn + 4Li_2_O directed the formation of the plateau around 0.4V.

As is shown in Figure 6B, charge and discharge tests were carried out between 0.0 and 3.0 V at the current density of 200 mA g^−1^ to evaluate the cycling stability of the prepared materials. There is little capacity loss on cycling stability except the first cycle. Like many other kinds of oxide anodes, the capacity increased during the cycle. After 110 cycles, the capacity is increased to ∼1,182 mA h g^−1^, while the Coulombic efficiency remained close to 100% after the fifth cycle, indicating that it had a very stable reversibility. Obviously, the nanosized Mn_3_O_4_ benefits the superior electrochemical performance.

The rate capacity of the naonosized Mn_3_O_4_ powders materials was showed in Figure 6C, at current rates of 200–10000 mA g^−1^. It could deliver capacities of 140 mA h g^−1^, and 40 mA h g^−1^, even at a superior high current density of 5,000 mA g^−1^ or 10,000 mA g^−1^, Apparently, the special rate capability benefits from the nanostructure, which provides a greatly reduced path for electron and ion transport.

**FIGURE 7:**
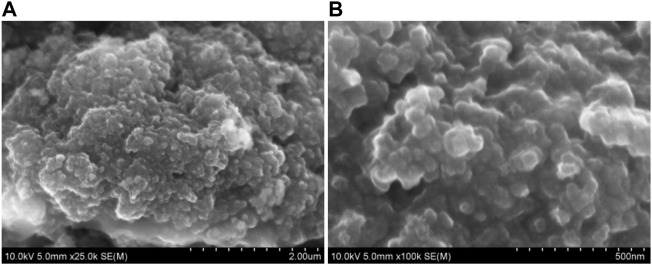
SEM images of nanosized Mn_3_O_4_ powders as anode materials after 110 cycles at a current density of 200 mA g^−1^: **(A)** micron scale, **(B)** nanometer scale.

In general, the nano structure improved the electrochemical performance of the material. The morphology of the materials after 110 cycles was investigated with SEM. As shown in Figure 7, the overall structure has not changed much after cycling. The results showed that the nanosized Mn_3_O_4_ is robust in structure, effectively reduced the structural strain and adapted to the large volume change in the process of repeated lithiation/delithiation.

## Conclusion

In summary, the nanosized Mn_3_O_4_ powder was prepared by flame spray pyrolysis. As an anode material for lithium-ion batteries, the Mn_3_O_4_ exhibits superior electrochemical performance in terms of stability and capacity. After 110 cycles, the Mn_3_O_4_ can still maintain a high capacity of more than 1,180 mA h g^−1^. The Mn_3_O_4_ has also a good rate performance, which is due to the nanosized features.

## Data Availability

The raw data supporting the conclusions of this article will be made available by the authors, without undue reservation.
